# Effect of Social Determinants of Health on Pediatric Traumatic Brain Injury Phenotypes: Retrospective Cohort Analysis

**DOI:** 10.2196/87372

**Published:** 2026-11-12

**Authors:** Adam Cross, Maanas Sandeep Agrawal, Anushree Udhayakumar, Paul M Arnold, Aaron T Anderson, Inki Kim

**Affiliations:** 1 Department of Pediatrics, Faculty of Medicine, University of Illinois College of Medicine Peoria, Peoria, IL, United States; 2 Coordinated Science Lab, Grainger College of Engineering, University of Illinois Urbana-Champaign, Urbana, IL, United States; 3 Information Management, School of Information Sciences, University of Illinois Urbana-Champaign, Urbana, IL, United States; 4 Neurological Surgery, Faculty of Medicine, Loyola Medicine, Maywood, IL, United States; 5 Beckman Institute for Advanced Science and Technology, University of Illinois Urbana-Champaign, Urbana, IL, United States; 6 Department of Biomedical and Translational Sciences, Faculty of Engineering and Medicine, University of Illinois Urbana-Champaign, 1206 W Clark St, CSL Studio, Urbana, IL, 16801, United States, 1 8147775676, 1 2173332003

**Keywords:** pediatric traumatic brain injury, Glasgow Coma Scale, social determinants of health, phenotypes, PhenoBench

## Abstract

**Background:**

Traumatic brain injury (TBI) in children disrupts cognitive, physical, and emotional functioning, posing a significant public health challenge in the United States. Recovery after pediatric TBI is nonlinear, influenced not only by fluctuations in progress but also by social determinants of health (SDoH), including sociodemographic features and health care access.

**Objective:**

This study investigates how SDoH influence TBI phenotypes and associated recovery trajectories. Our retrospective cohort analysis evaluates health disparities among pediatric patients and examines how these disparities influence health outcomes following TBI.

**Methods:**

We extracted 926 pediatric patients from the Children’s Health After Injury (CHAI) dataset to ensure consistency across 5 key variables: Abbreviated Injury Scale (AIS) scores, vital signs and assessments, comorbidities, complications, and Glasgow Coma Scale (GCS) scores. Dimensionality reduction and density-based hierarchical clustering identified distinct phenotypes and their SDoH characteristics. Further exploratory analysis revealed distinct recovery trajectories across the phenotypes. To confirm the effect of SDoH on the phenotypes, generalized linear modeling and the random forest modeling were used.

**Results:**

Four distinct pediatric TBI phenotypes were identified, each with a unique injury-severity profile, recovery trajectory, and pattern of SDoH association: (1) adolescents with high injury severity and stable GCS scores (n=278) showed a significant association between phenotype membership and age. This group experienced a gradual increase in psychological and behavioral impairment over the recovery period, with total impairment scores increasing by 25% from baseline to the 12-month follow-up. (2) Younger children with moderate injuries and stable GCS scores (n=141) showed no significant SDoH associations. Total impairment scores remained stable across the follow-up period, though neurological and physical conditions showed long-term worsening, warranting ongoing monitoring. (3) Critically injured children with severe neurological impairment (n=98) showed the strongest SDoH associations, with race/ethnicity and its interaction with age significantly linked to phenotype membership; Asian children had 1.8 (95% CI 1.02‐3.34) times the odds of belonging to this critically injured phenotype compared with White children. Despite carrying the lowest overall impairment of any cluster at baseline, this group showed escalating psychological burden and fluctuating neurological conditions through the 12-month follow-up. (4) Moderately injured children with stable GCS scores followed by a sharp progression (n=409) showed no significant SDoH associations, yet carried the highest overall impairment burden of any cluster across all time points. Total scores increased 31% from baseline to the 12-month follow-up, driven by a marked escalation in psychological/neurological issues.

**Conclusions:**

Age and race/ethnicity were the most influential predictors of cluster membership, with SDoH risk concentrated in the 2 most severely injured groups. Identified SDoH-driven phenotypic disparities can guide targeted interventions for pediatric patients at high risk for TBI sequelae. Future studies should extend follow-up beyond 12 months and validate these phenotypes in diverse cohorts to strengthen generalizability and inform equitable care.

## Introduction

### Background: Traumatic Brain Injury Prevalence in the Pediatric Population

Pediatric traumatic brain injury (TBI) continues to be a leading cause of morbidity and mortality among children in the United States [1]. Approximately 50,000 children are hospitalized annually due to TBI, highlighting its substantial health care and societal burden [2]. A 2020 epidemiological study estimated that 6.8% of children have experienced symptoms of mild TBI (ie, concussion), and its prevalence tends to increase with age, particularly among adolescents [3]. Children who sustain concussions are generally at risk of long-term cognitive, physical, and behavioral impairments that adversely affect their academic performance, social relationships, and overall quality of life [4]. Approximately 145,000 children and adolescents aged 0 to 19 years are currently living with prolonged impairments due to TBI [4].

### Prior Work on Health Disparities and Socioeconomic Factors in Pediatric TBI

Significant disparities exist in the reporting and outcomes of TBI influenced by race/ethnicity, sex, and the socioeconomic status of pediatric patients [2]. Ethnic minorities often encounter barriers to health care, leading to underreporting of symptoms and suboptimal patient outcomes [5]. Male individuals are generally at a higher risk of experiencing TBI symptoms compared to female individuals due to their greater participation in high-risk activities [3]. Another contributing factor is that children from families with lower socioeconomic status are less likely to access specialized TBI care and rehabilitation services, resulting in delayed diagnoses and inadequate long-term management [6].

Social determinants of health (SDoH) [7] refer to nonmedical factors influencing health outcomes, such as economic stability, ethnicity, health care access, and the local community. SDoH have been reported to impact pediatric TBI. Socioeconomic constraints often impede access to quality care, delaying timely diagnoses and resulting in poorer outcomes in TBI [6]. For instance, residing in rural areas may reduce access to specialized trauma care [8]. Children’s insurance and health literacy are also known to affect their (or their parents’) abilities to navigate medical resources and secure necessary services to support recovery from pediatric TBI [9]. Additionally, unsafe living environments and the lack of safe recreational areas may further increase the incidence of TBI and hinder rehabilitation efforts [10].

While prior research has explored the impact of SDoH on adult TBIs [11], there is a paucity of studies focusing specifically on pediatric TBI. Consequently, SDoH and health equity have been significantly underrepresented as crucial variables in developing clinical guidelines and recommendations for TBI diagnosis and treatment [12]. The TBI literature has identified several key factors that influence patient outcomes, including race, ethnicity, insurance status, socioeconomic status, gender, age, access to health care, geographic location, and medical comorbidities [11]. However, comprehensive analyses that specifically examine how these factors shape and influence patient clusters across recovery trajectories are limited [2]. This knowledge gap underscores the need for this research and the development of targeted interventions to manage pediatric TBI effectively.

### Goal of This Study

This study aims to identify marginalized pediatric groups that are more susceptible to poor health outcomes following TBI due to disparities in SDoH. Specifically, the study seeks to develop pediatric TBI “phenotypes” [13] (ie, distinct patient clusters based on injury characteristics) and analyze how those clusters interact with SDoH to influence recovery and long-term management.

Hypothetically, SDoH will significantly impact the phenotypic manifestations of TBI in children. Assuming that distinct TBI phenotypes are identified, this retrospective cohort analysis will characterize significant differences in their severity and recovery trajectories and interpret the effects of adverse SDoH, such as lower household income and differences in health insurance coverage, compared with those associated with higher socioeconomic status.

Investigating the hypothetical relationships between SDoH and TBI phenotypes and their impact on recovery is useful for identifying at-risk populations and developing targeted interventions. Furthermore, it will enhance health care delivery by refining screening protocols tailored to minority pediatric populations, ensuring timely care for all children. At last, the insights gained from this study will inform health care policy and protocols, enabling policymakers to address health disparities toward equitable outcomes.

## Methods

### Study Design

This study uses a retrospective cohort analysis [14] of the Children’s Health After Injury (CHAI) dataset [15], one of the largest prospective longitudinal studies on pediatric TBI, designed to determine population-based rates of disability after TBI in children and adolescents, with an outstanding 3-year follow-up. The original CHAI study recruited a cohort of children aged 0 to 18 years who were discharged from the emergency department (ED) or admitted as inpatients and treated for a TBI at the Children’s Hospital of Philadelphia, Seattle Children’s Hospital, Harborview Medical Center (a level 1 trauma center in King County, Washington), and four level 3 and one level 4 trauma centers randomly sampled in King County, with enrollment occurring between March 1, 2007, and September 30, 2008. Outcomes of the CHAI study were tracked longitudinally against preinjury baseline at multiple follow-up points measuring disability broadly in health-related quality of life, adaptive skills, and participation in social and community activities at 3, 12, and 24 months postinjury, with related publications extending follow-up to 36 months. This multisite, multiyear structure forms the basis of this study to draw our subset and identify clusters, and analyze the effects of SDoH on those clusters.

CHAI itself meets the rigorous standard of cohort design requirements [14] as a solid representation of the source population in the United States, via a prospective, multisite design with a fixed enrollment window. In comparison, the cohort and subclusters of this study are a retrospective rederivation from that resource. Distinct from CHAI’s original exposure-based recruitment, we selected patient subsets for “completeness” across key clinical variables, including Abbreviated Injury Scale (AIS), Glasgow Coma Scale (GCS) [16], and vital signs, which is a complete-case selection strategy (rather than an exposure-based one). This is a meaningful design shift that, according to the standard cohort design framework [14], warrants its own representativeness check.

The CHAI study design is well suited to examining our hypothesis, as it allows exploratory analysis of TBI phenotyping over recovery trajectories. Specifically, we incorporated additional health features, including medical histories of hearing and visual impairment (Table 1), to map the recovery trajectories of each cluster from baseline through the 3-month and 12-month follow-up visits. The baseline and follow-up data will henceforth be referred to as “longitudinal data.”

Subsequently, we fit individual generalized linear models (GLMs) for each phenotype, with SDoH variables such as race, ethnicity, and gender as predictors to elucidate how they influence the classification of data points into each phenotype; see Figure 1 for the study design.

**Table 1.** Variables/features used to evaluate the impact of social determinants of health (SDoH) on phenotype characterization and recovery.

Variable	Definition	Type and scale	Assessment method
Clinical measures used to distinctly categorize patients into phenotypes (independent variables)			
Injury assessment	Abbreviated Injury Scale (AIS): classifies severity of traumatic injuries across multiple body regions	Mixed (AIS: 1‐5 per body region)	Clinician-assessed
Vital signs	ED^a^ and ICU^b^ measurements, including respiratory rate, heart rate, blood pressure, and oxygen saturation. Supplemental measures include temperature, respiration type, circulatory support, and arterial gas pressures	Mixed (varies by element)	Clinician-measured
Comorbidities	Preexisting conditions documented via general health history (eg, ADHD^c^, seizure history, and learning disabilities) and behavioral history (eg, substance use), through clinician-facilitated interview	String	Clinician-reported and self-reported
Complications	Postinjury complications (second insults), including seizures, hypotensive or hypoxic episodes, cardiac arrest, electroencephalogram (EEG) monitoring, and hyperventilation	String	Clinician-reported
Glasgow Coma Scale (GCS) Score	Standardized TBI^d^ consciousness assessment across 3 domains: eye opening, verbal response, and motor response. Recorded pre-ED, in-ED, and at discharge	Integer, 3 to 15	Clinician-administered
Sociodemographic features used to derive insights on SDoH (covariates)			
Age	Age of the patient	Integer (years)	Self-reported by patient/caregiver
Gender	Gender of the patient	String (male, female, unspecified, unreported, and unknown)	Self-reported by patient/caregiver
Household size	Number of household members	Integer (≥1)	Self-reported by patient/caregiver
Marital status	Marital status of the patient’s parent or caregiver	String	Self-reported by patient/caregiver
Race/ethnicity	Patient’s racial and ethnic background	String	Self-reported by patient/caregiver
Employment status	Employment status of the patient’s parent or caregiver	String	Self-reported by patient/caregiver
Health insurance type	Type of health insurance coverage	String	Self-reported by patient/caregiver
Household income	Annual household income	Integer (≥0)	Self-reported by patient/caregiver
Health and medical history for recovery: psychological and behavioral markers			
Mental health	Quality of life in neurological disorders (Neuro-QOL): patient-reported outcome system for neurologically impaired populations. Comprises 12 Likert-based item banks across domains, including depression, anxiety, cognitive function, fatigue, and sleep disturbance, with patient-reported outcomes measurement information system (PROMIS) equivalency scoring	Integer (Likert; lower=better mental health; higher=poorer mental health)	Self-reported by patient
Anxiety	SCARED (Screen for Child Anxiety Related Emotional Disorders): 41-item child self-report measure with 5 subscales aligned to *DSM-IV* (*Diagnostic and Statistical Manual of Mental Disorders* [Fourth Edition]) anxiety classifications. A score of 25 or above indicates clinically significant anxiety	Integer, 0 to 82	Self-reported by patient
Depression	Center for Epidemiologic Studies Depression Scale (CES-D): 20-item scale covering 6 symptom domains, including depressed mood, guilt, hopelessness, psychomotor retardation, appetite loss, and sleep disturbance. Items rated 0 to 3; 4 items are reverse-scored	Integer, 0 to 60	Self-reported by patient
Attention	Strengths and Difficulties Questionnaire (SDQ): 25-item behavioral screener with 5 subscales: emotional symptoms, conduct problems, hyperactivity/inattention, peer problems, and prosocial behavior. Completed by children aged 11 to 16 years or by parents/teachers of children aged 4 to 16 years	Integer, subscale 0 to 10; total 0 to 40	Parent/caregiver-reported
Behavioral	Child Behavior Checklist (CBCL): parent/caregiver-reported measure in 2 age-specific forms (CBCL/1.5‐5 and CBCL/6‐18). Assesses 3 competence domains: activities, social, and school	Integer, T-score (mean 50, SD 10)	Parent/caregiver-reported
Health and medical history for recovery: developmental and sensory markers			
Developmental delay	Bayley Scales of Infant Development (Bayley-III/BSID), for children aged 1 to 42 months. Clinician-administered: cognitive, language, and motor scales. Parent/caregiver-completed: social-emotional and adaptive behavior scales	Integer (BSID-III: 0/1; BSID-4: 0/1/2)	Clinician-administered and parent/caregiver-reported
Speech problems	Words in Noise Test (WIN): assesses speech perception in competing noise using monosyllabic words at 7 signal-to-noise ratios (0‐24 dB). Part of the National Institute on Deafness and Other Communication Disorders (NIDCD) voice, speech, and language domain	Integer, 0 to 100 (% correct word recognition)	Clinician-assessed
Hearing impairment	Hearing Handicap Inventory (HHI): 25 items assessing degree of handicap due to hearing problems (emotional and social/situational subscales). Part of the NIDCD hearing domain	Integer, 0 to 100	Self-reported
Vision impairment	National Institutes of Health (NIH) Toolbox sensory battery: evaluates vision function across cognitive, emotional, sensory, and motor domains. Lower scores indicate poorer visual ability	Integer (varies by test)	Clinician-assessed
Health and medical history for recovery: neurological and physical condition markers			
Chronic pain	Assessed via Neuro-QOL; see “mental health” variable for instrument details	Mixed (Likert; lower=better outcome)	Self-reported by patient
Sleep disturbances	Assessed as a CES-D subscale; see “depression” variable for instrument details	Mixed (Likert)	Self-reported by patient
Epilepsy	Diagnosis reported by patient/caregiver and confirmed via medical record. Also assessed via Neuro-QOL (see “mental health” variable)	Mixed	Self-reported by patient/caregiver

a ED: emergency department.

b ICU: intensive care unit.

c ADHD: attention-deficit/hyperactivity disorder.

d TBI: traumatic brain injury.

**Figure 1.**
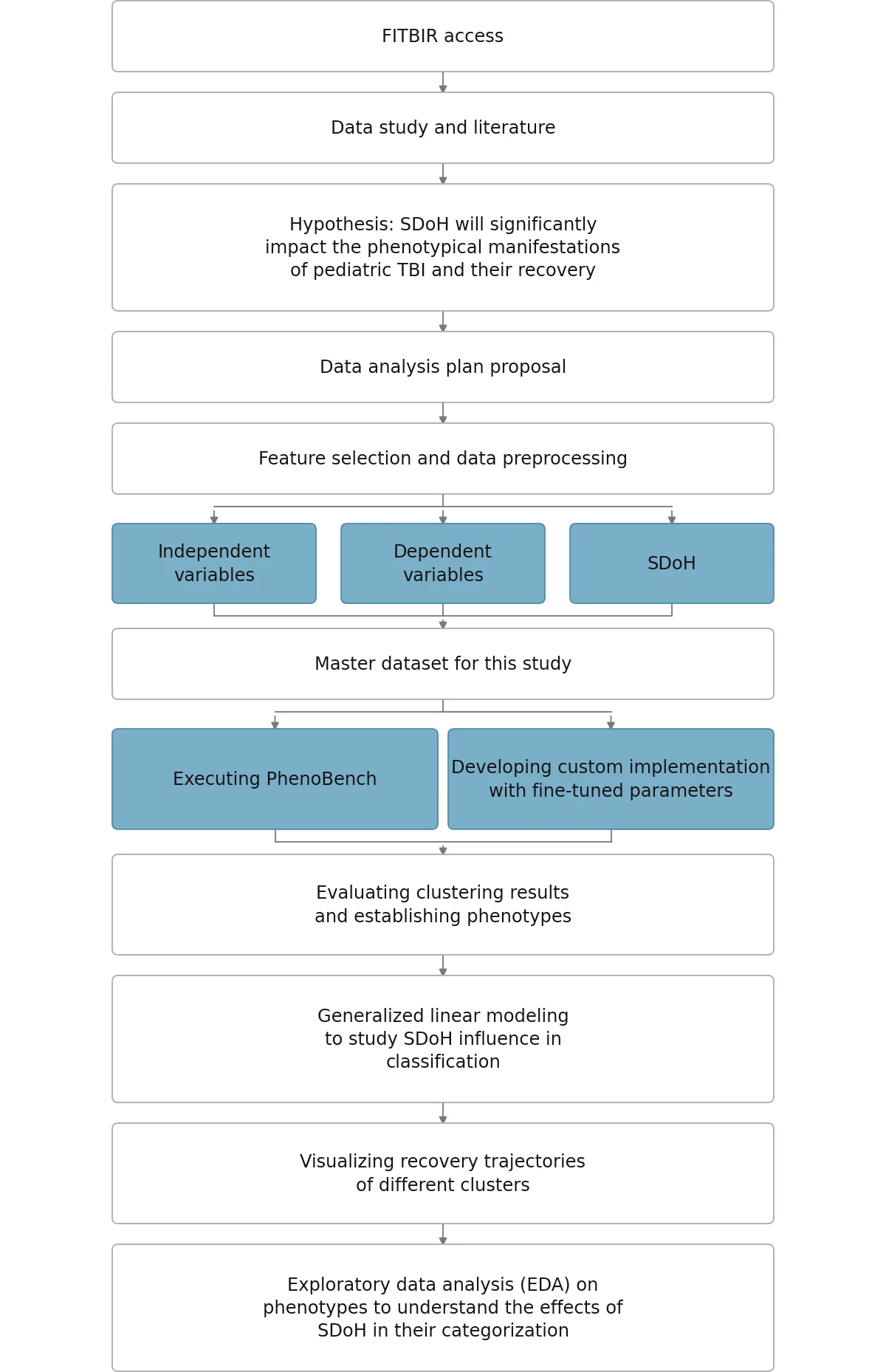
Study design. FITBIR: Federal Interagency Traumatic Brain Injury Research; SDoH: social determinants of health; TBI: traumatic brain injury.

### Ethical Considerations

Access to the dataset was granted (application number 117428, approved on April 16, 2024), and the data were analyzed using the controlled datasets distributed from the Department of Defense (DoD)–supported and National Institutes of Health (NIH)–supported FITBIR (Federal Interagency Traumatic Brain Injury Research) Informatics System. FITBIR is a collaborative biomedical informatics system created by the Department of Defense and the NIH to provide a national resource to support and accelerate research in TBI.

### PhenoBench for Phenotyping Standardization and Clustering of Pediatric TBI

PhenoBench is a tool developed by Pugh et al [17] to standardize phenotyping across studies, offering a robust framework for clustering and dimensionality reduction. It adopts uniform manifold approximation and projection and principal component analysis (PCA) [18] for dimensionality reduction, and implements K-means and HDBSCAN (hierarchical density-based spatial clustering of applications with noise) [19] algorithms for clustering, which ensure consistent, reproducible, and clinically actionable results.

#### Dimension Reduction

Dimensionality reduction is performed because high-dimensional datasets, such as the 72-feature CHAI dataset used in this study, are difficult to visualize and interpret directly. PhenoBench applies uniform manifold approximation and projection and PCA to reduce this feature space into 2 principal components: PCA1 and PCA2. Each principal component is a linear combination of the original variables derived from the eigenvectors of the data covariance matrix. PCA1 captures the axis of greatest variance in the dataset; PCA2 captures the next greatest variance, constrained to be orthogonal to PCA1. Together, they form a two-dimensional projection that retains the most informative structure of the original feature space, facilitating a better understanding of the distribution of TBI phenotypes. In simple terms, PCA1 and PCA2 are the 2 axes that best summarize the variation across patients: PCA1 points in the direction where patients differ from each other the most, and PCA2 captures the next most meaningful direction of difference, independent of PCA1. Plotting patients along these 2 axes allows clinically distinct groups to become visually apparent.

#### Clustering

For benchmarking, we used PhenoBench on the curated CHAI dataset to commence our analysis, and then, for comparison, implemented custom K-means and HDBSCAN algorithms. The results of HDBSCAN (Figure S1 in Multimedia Appendix 1) and K-means clustering were compared against PhenoBench’s output to ensure alignment.

Our custom implementation enabled us to adapt PhenoBench’s clustering methods to better align with the characteristics of the CHAI dataset, offering enhanced flexibility for exploration and optimizing computational efficiency. This customized approach had several practical advantages over PhenoBench. First, our custom K-means was particularly well-suited to our dataset for producing clusters (Figure 2), helping identify distinct phenotypes and meaningful subgroups. Second, handling multiple datasets in CSV format necessitated iterative feature selection to test hypotheses and address inconsistencies, such as empty variables, which required multiple iterations. PhenoBench, while robust, offered limited flexibility for iterative adjustments and necessitated complete execution of the tool, resulting in increased computational overhead.

For performance comparison, PhenoBench automatically determined the number of clusters to be 4 and achieved a silhouette score of 0.4693, indicating well-defined clusters with strong separation. Our custom clustering approach produced a comparable score of 0.4415, also reflecting satisfactory clustering quality, albeit slightly lower than that of PhenoBench.

**Figure 2.**
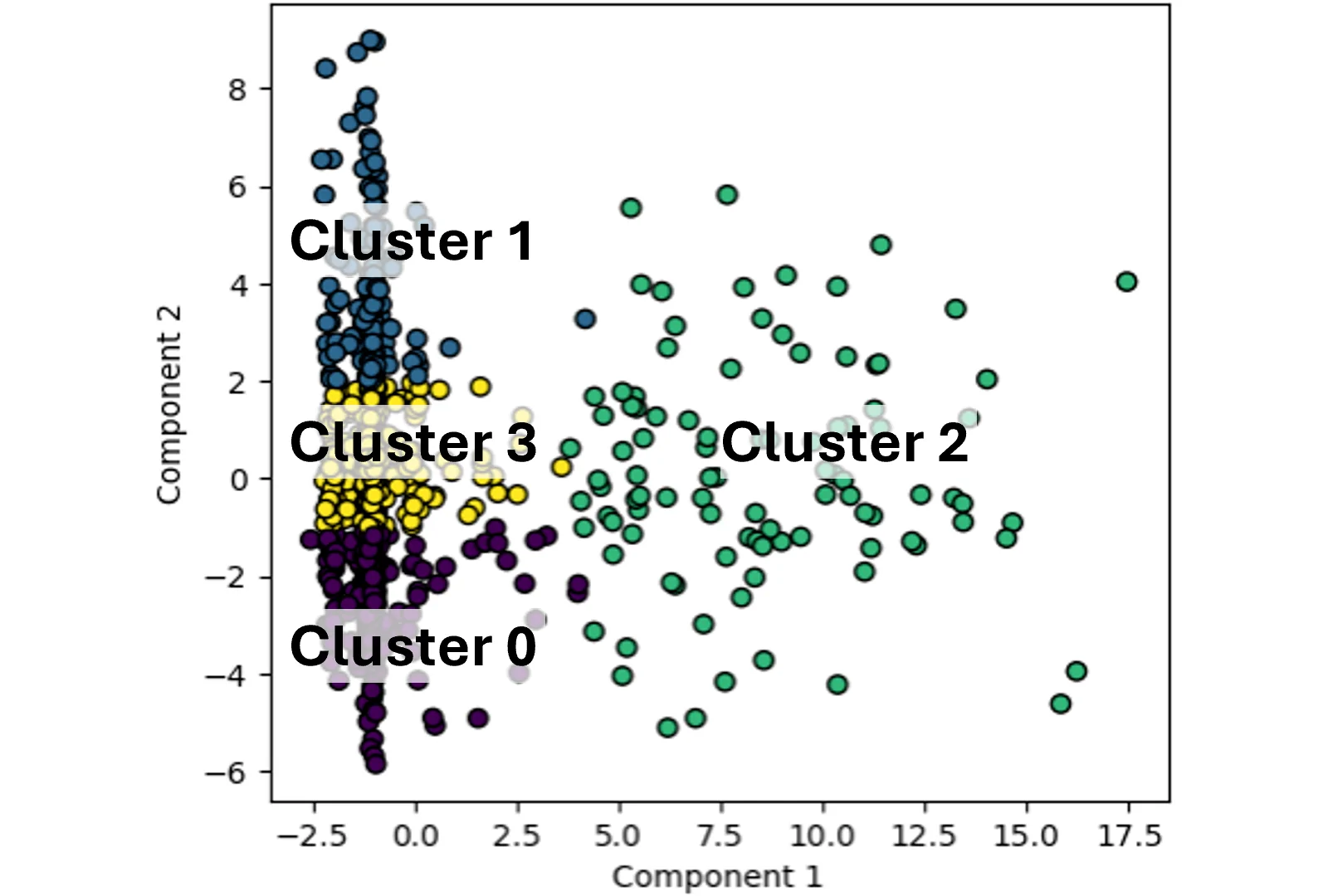
Custom principal component analysis (PCA) with K-means clustering generated 4 patient clusters.

### Exploratory Analysis

We further explored the evolution of fundamental data points, including the average GCS score, injury severity, and age, to help delineate the clusters. As the primary axes in the plots are PCA1 and PCA2 (ie, components 1 and 2 in Figure 2), we lack a clear understanding of how the 72 features have contributed to the clustering of the data points. Consequently, we undertook a reverse-engineering approach to extract pertinent information, thereby elucidating the underlying significance of the resulting 4 clusters.

## Results

### The 4 Clusters (Phenotypes) of Pediatric TBI

#### Cluster 0: Adolescents With High Injury Severity and Stable GCS (n=278)

This cluster comprises 278 patients with an average age of 14.41 (SD 2.72) years (Table 2). This group presents the most severe head injuries, characterized by a mean head AIS score of 2.14 (SD 1.47). However, they maintain stable mean GCS scores of 14.81 (SD 0.63), suggesting preserved consciousness despite substantial trauma. The low intubation rate of 2.52% (n=7) aligns with these stable neurological outcomes. Adolescents in this cluster may have benefited from age-related resilience, contributing to their ability to maintain high functional status despite comparable injury severity.

**Table 2.** Summary of social determinants of health (SDoH) features and each of their distributions across the phenotypes.

Cluster characteristics	Cluster 0	Cluster 1	Cluster 2	Cluster 3
Number of patients, n	278	141	98	409
Age of patients (year), mean (SD)	14.41 (2.72)	5.63 (2.92)	8.80 (5.99)	7.90 (4.64)
Abbreviated Injury Scale, mean (SD)	2.14 (1.47)	1.80 (1.10)	1.85 (1.23)	2.07 (1.35)
Glasgow Coma Scale Score, mean (SD)	14.81 (0.63)	14.90 (0.64)	9.56 (3.24)	14.81 (0.70)
Pseudo-*R*^2^	0.01476	0.0085	0.0216	0.0067
Race/ethnicity, n (%) within each cluster				
White	174 (62.59)	84 (59.57)	65 (66.33)	251 (61.37)
Hispanic or Latino	30 (10.79)	10 (7.09)	10 (10.20)	46 (11.25)
Black or African American	10 (3.60)	10 (7.09)	1 (1.02)	18 (4.40)
Asian	2 (0.72)	7 (4.96)	1 (1.02)	10 (2.44)
Sex of patient, n (%) within each cluster				
Male	179 (64.39)	99 (70.21)	59 (60.20)	263 (64.30)
Female	92 (33.09)	42 (29.79)	33 (33.67)	141 (34.47)
Parent’s employment status, n (%) within each cluster				
Working full/part time	164 (58.99)	89 (63.12)	54 (55.10)	241 (58.92)
Full-time homemaker	67 (24.10)	35 (24.82)	21 (21.43)	104 (25.43)
Not working	14 (5.04)	2 (1.42)	5 (5.10)	13 (3.18)
Not working for studying	9 (3.24)	3 (2.13)	3 (3.06)	6 (1.47)
Patient health insurance type, n (%) within each cluster				
Insurance	170 (61.15)	95 (67.38)	59 (60.20)	261 (63.81)
Medicaid/Disproportionate Share Hospital (DSH) Payment	57 (20.50)	32 (22.70)	23 (23.47)	96 (23.47)
Others (specified)	24 (8.63)	9 (6.38)	3 (3.06)	21 (5.13)
No insurance	13 (4.68)	2 (1.42)	2 (2.04)	16 (3.91)
Parents’ household income (US $), n (%) within each cluster				
<20,000	24 (8.63)	18 (12.77)	15 (15.31)	50 (12.22)
20,001‐30,000	36 (12.95)	7 (4.96)	10 (10.20)	37 (9.05)
30,001‐40,000	22 (7.91)	11 (7.80)	4 (4.08)	29 (7.09)
>100,001	86 (30.94)	44 (31.21)	32 (32.65)	139 (33.99)
Parents’ marital status, n (%) within each cluster				
Divorced	29 (10.43)	17 (12.06)	13 (13.27)	41 (10.02)
Married	187 (67.27)	97 (68.79)	63 (64.29)	267 (65.28)
Not married	27 (9.71)	13 (9.22)	9 (9.18)	51 (12.47)
Remarried	7 (2.52)	4 (2.84)	4 (4.08)	7 (1.71)
Separated	13 (4.68)	7 (4.96)	7 (7.14)	24 (5.87)
Widowed	4 (1.44)	0 (0)	1 (1.02)	5 (1.22)

#### Cluster 1: Younger Children With Moderate Injuries and Stable GCS (n=141)

This cluster comprises 141 patients, with an average age of 5.63 (SD 2.92) years, representing the youngest group among all clusters. These patients exhibit moderate head injury severity, with a mean head AIS score of 1.80 (SD 1.10) and a stable mean GCS score of 14.90 (SD 0.64). The intubation rate was remarkably low (n=2, 1.42%), indicating that most patients retained sufficient neurological function and did not require critical airway management. Despite their younger age and potential vulnerability, these patients demonstrate favorable outcomes, possibly attributable to the lesser severity of their injuries.

#### Cluster 2: Critically Injured Patients With Severe Neurological Impairment (n=98)

This cluster comprises 98 critically injured patients, with an average age of 8.80 (SD 5.99) years. This group exhibits significantly lower GCS scores, with a mean of 9.56 (3.24), indicating moderate to severe neurological impairment. Notably, despite the lower mean head AIS score of 1.85 (SD 1.23) compared to cluster 3, the neurological status of these patients is more compromised. This discrepancy suggests that injury mechanisms or non–head-related systemic factors may contribute to poorer outcomes in this group.

#### Cluster 3: Moderately Injured Patients With Stable GCS (n=409)

This cluster comprises 409 patients with an average age of 7.90 (SD 4.64) years. This group exhibits moderate head injury severity, as evidenced by a mean head AIS score of 2.07 (SD 1.35). Concurrently, they demonstrate stable consciousness levels, as indicated by a mean GCS score of 14.81 (SD 0.70). The intubation rate was 1.22% (n=5), suggesting that most patients did not necessitate invasive airway management. These findings collectively indicate that, while injuries are not insignificant, patients within this cluster require minimal clinical interventions.

### Clinical Interpretation: AIS-GCS Correlation Within Each Cluster

Correlations between AIS and GCS were computed across 3 GCS measurement time points (initial ED assessment, during the ED stay, and at ED discharge). Values reported below represent a range that reflects how the AIS-GCS relationship evolves from admission through discharge, rather than a single static coefficient.

Across clusters, only cluster 2 showed a clinically meaningful negative AIS-GCS correlation, consistent with dominant cranial injury driving consciousness impairment. The remaining 3 clusters exhibited weak or near-zero correlations, indicating that for these phenotypes, a higher AIS score does not reliably lower GCS score, reflecting a predominance of noncranial injuries, mild cranial involvement, or the effects of prompt clinical stabilization. These findings confirm that AIS is a robust predictor of GCS only where serious head injuries dominate.

In cluster 0 (adolescents with high injury severity and stable GCS), the AIS-GCS correlation ranged from approximately –0.15 at initial ED assessment to –0.07 at discharge and was consistently weak and negative. Although the mean maximum head AIS score is approximately 2.21 (SD≈1.47), the majority of patients maintained a high GCS score (mean≈14.90, SD≈0.63), likely because injuries are mixed or extracranial, diluting a direct severity-consciousness relationship. In cluster 1 (younger children with moderate injuries and stable GCS scores), the correlation is weakly positive across time points (+0.06 to +0.12), a paradoxical pattern explained by the predominance of noncranial injuries (eg, limb fractures) in which higher AIS score does not impair neurological function.

Cluster 2 (critically injured patients with severe neurological impairment) showed the strongest and most clinically significant AIS-GCS relationship, deepening from –0.30 at initial assessment to –0.54 at discharge. This trajectory reflected progressive confirmation of cranial injury severity over time. Despite a mean head AIS score of 1.96, patients in this cluster had a mean daily GCS score of only 9.55, indicating sustained neurological compromise that did not resolve before discharge and distinguishing this group from the other three phenotypes.

Cluster 3 (moderately injured patients with stable GCS) exhibited near-zero correlations throughout (ranging from +0.028 at initial assessment to +0.050 at discharge), despite a mean head AIS score of 2.14 (SD 1.47). The preserved mean GCS score of 14.86 suggested that injuries in this cluster were largely noncritical or that prompt stabilization prevented AIS-level changes from translating into measurable consciousness impairment.

### Longitudinal Analysis of Clusters Over Recovery

We further examined various impairment issues, including mental health problems (anxiety, depression, attention problems, and behavioral issues), chronic pain, developmental delays, hearing impairments, sleep disturbances, speech problems, vision issues, and epilepsy, across temporal progression. The evaluation took place at 3 different points in time: before injury or baseline, follow-up after 3 months, and follow-up after 12 months (FU12). This longitudinal analysis reveals differential recovery trajectories across phenotypes (Figure 3). For more details, see Figures S7 to S9 in Multimedia Appendix 1 for the cluster-wise recovery trajectories stratified by specific impairment categories (physiological, behavioral, developmental, sensory, neurological, and physical).

*Cluster 0*, despite maintaining stable GCS score throughout, carried a progressively worsening psychological and behavioral burden, with impairment scores rising from baseline to FU12. Developmental and sensory impairments follow a nonlinear pattern: an initial decline followed by a late increase, pointing to delayed but real sensory consequences. Neurological and physical conditions also trend upward, consistent with ongoing pain or sleep disturbance. The disconnect between acute stability and long-term psychological deterioration underscores the need for sustained psychological follow-up in this adolescent cohort.

*Cluster 1* shows the most favorable recovery trajectory of the 4 phenotypes. Psychological and behavioral issues remain stable or show only minimal increases across time points, while developmental and sensory impairments follow a consistent downward trend, suggesting effective sensory adaptation in this youngest cohort. Neurological and physical conditions rise modestly through 3 months but plateau thereafter, pointing to manageable chronic challenges, such as mild pain, rather than progressive neurological deterioration. These findings are reassuring, but they support continued neurological monitoring, given the young age at injury.

*Cluster 2* exhibits the most severe and unrelenting longitudinal trajectory: psychological and behavioral issues rise consistently through FU12, reflecting sustained cognitive and emotional burden commensurate with this group’s acute neurological compromise. Unlike in other clusters, neurological and physical conditions fluctuate rather than stabilize, indicating complex and variable symptom profiles (including epilepsy and chronic pain) that resist simple recovery patterns. Developmental and sensory impairments show only minimal decline, offering little compensatory improvement. Comprehensive, multidisciplinary rehabilitation addressing both neurological and psychological domains is critical for this group.

**Figure 3.**
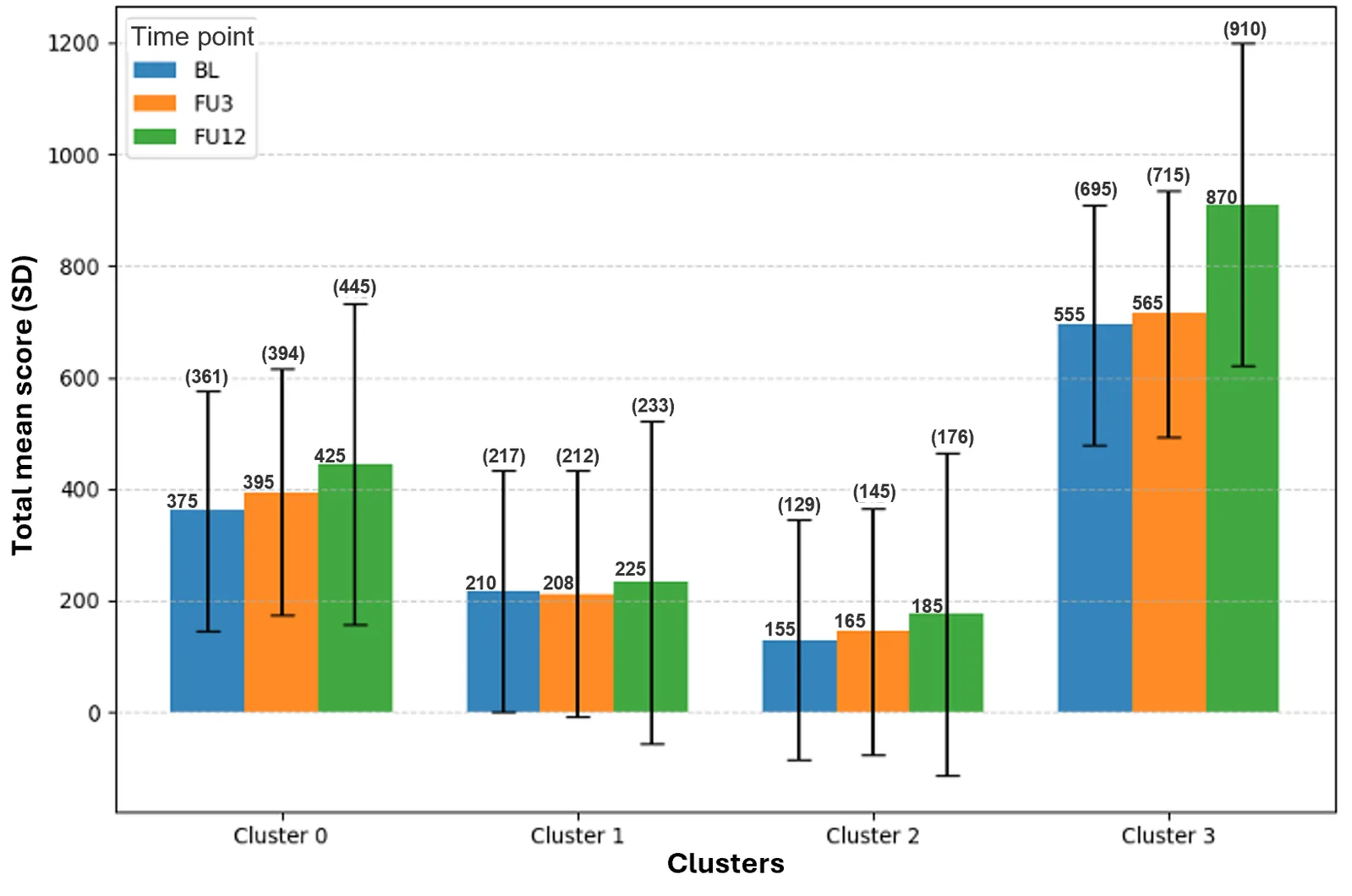
Overall impairment scores (total mean score, SD) representing health and medical history for each cluster across 3 different time points (baseline [before injury], FU3, and FU12): cluster 3 consistently shows across time the highest scores (baseline [before injury]=695, FU3=715, and FU12=910), indicating the greatest overall impairment recovery (?) compared with the other clusters; cluster 0 follows next (361-445), while clusters 1 and 2 remain substantially lower throughout; the variable-length whiskers (SD≈150‐300) indicate considerable variability in impairment scores across clusters, with recovery trajectories diverging most strongly at FU12. FU3: follow-up after 3 months; FU12: follow-up after 12 months.

*Cluster 3* reports a steep increase in psychological and behavioral issues, particularly between 3 and 12 months postinjury, indicating worsening mental health outcomes over time. However, this group also shows a notable decline in developmental and sensory impairments, highlighting successful sensory adaptation. Conversely, neurological and physical conditions increase sharply, suggesting escalating issues related to pain or neurological symptoms. This trajectory reflects delayed psychological and neurological challenges, underscoring the importance of ongoing mental health support and effective pain management strategies. Notably, despite being characterized by moderate injury severity and low acute intervention needs, cluster 3 carries the highest overall impairment burden across all time points (Figure 3); this divergence suggests that injury-independent psychosocial stressors drive the escalating trajectory and warrant prospective investigation.

### Statistical Modeling of Relationships Between SDoH and TBI Phenotypes

To examine how SDoH influence pediatric TBI phenotypes, we conducted 2 analyses. First, we assessed the overall sociodemographic composition of phenotypes using multinomial logistic regression, with cluster 0 as the reference category. This model included race/ethnicity, gender, household income, and insurance type as predictors. Race/ethnicity was collapsed into 5 mutually exclusive categories (White [reference], Black/African American, Hispanic/Latino, Asian, and Other/Mixed group) and dummy-coded. We tested global differences using a chi-squared test (α=.05) and examined specific group differences using pairwise Wald contrasts, applying a Bonferroni correction to control for multiple comparisons.

Although the global distribution of race/ethnicity across clusters was not statistically significant (*χ*²_12_=16.4, *P*=.17), the multinomial logistic regression revealed significant associations for the Asian group. Compared with White children, Asian children had 5.2 (95% CI 1.31‐20.6) times the odds of being in cluster 1, and 1.8 (95% CI 1.02‐3.34) and 2.6 (95% CI 1.15‐5.76) times the odds of being in clusters 2 and 3, respectively (Table 3). No significant associations were found for Black, Hispanic/Latino, or Other/Mixed groups relative to White group.

Second, we fitted 4 binary GLMs, 1 for each cluster, by treating the corresponding phenotype as the positive class and all others as the negative class. These models included the full set of SDoH predictors and interaction terms to identify variables specifically associated with individual phenotype membership. For the summary of SDoH features and their distributions among the phenotypes, see Table 2. Note that the percentages indicative of within-cluster distributions were computed for each cell, dividing each frequency by that of the corresponding cluster's subtotal (rather than the overall total of 926).

**Table 3.** Comparative analysis of the odds of cluster membership by race/ethnicity.

Ethnicity (reference: White)	Cluster 1, OR^a^ (95% CI)	Cluster 2, OR (95% CI)	Cluster 3, OR (95% CI)	Statistical significance
Black	1.6 (0.63‐4.17)	0.23 (0.03‐1.84)	0.46 (0.19‐1.10)	Not significant
Hispanic/Latino	0.57 (0.25‐1.30)	0.52 (0.17‐1.57)	0.88 (0.40‐1.91)	Not significant
Asian	*5.2* (1.31‐20.6)	1.8 (1.02‐3.34)	2.6 (1.15‐5.76)	Significant
Other/Mixed	1.15 (0.68‐1.93)	0.68 (0.30‐1.55)	1.26 (0.73‐2.19)	Not significant

a OR: odds ratio.

The results of the cluster-specific binary GLMs are summarized below:

- *Cluster 0*: The model had limited explanatory power (pseudo-*R*²=0.0148). A significant positive interaction was found between age group and race/ethnicity (coefficient=0.0807, *P*=.03), suggesting these variables jointly influence phenotype membership. Parental status was marginally associated (*P*=.07).
- *Cluster 1*: The model also showed low explanatory power (pseudo-*R*²=0.0085). No predictors reached statistical significance (all *P*>.05), suggesting that SDoH variables had limited influence on this group.
- *Cluster 2*: Model fit improved slightly (pseudo-*R*²=0.0216). Significant associations were found for race/ethnicity (coefficient=0.4048, *P*=.001) and its interaction with age group (coefficient=–0.1437, *P*=.003), indicating a nuanced, age-modulated racial disparity. The age variable showed a marginal effect (*P*=.05).
- *Cluster 3*: The model showed minimal explanatory power (pseudo-*R*²=0.0067). None of the SDoH variables reached significance, suggesting that factors outside the measured demographic and socioeconomic domains may contribute more strongly to this phenotype.

Taken together, the SDoH signal is concentrated in clusters 0 and 2, suggesting that race/ethnicity (including its interaction with age) are the primary demographic drivers of phenotype membership in pediatric TBI, and that clinically stable presentations can still carry meaningful sociodemographic risk.

To further evaluate the importance of individual variables, a random forest model (scikit-learn RandomForestClassifier) was used as a confirmatory tool that supported the observed associations. Feature importance is quantified using mean decrease in impurity: at each split node, the weighted reduction in Gini impurity attributable to the splitting variable is recorded; scores are averaged across all trees in the forest and normalized to sum to 1, so each value represents that variable’s proportional contribution to separating patients into distinct phenotype classes. The random forest in Figure 4 echoes the significance of age as the most influential predictor of phenotype classification, highlighting its pivotal role in shaping recovery patterns. Additionally, race and ethnicity were found to be significant factors, corroborating previous research that emphasized the importance of demographic considerations. Socioeconomic variables, such as income and parental education, exhibited moderate influence, demonstrating their continued contribution to phenotypic profiles; Figure S3 in Multimedia Appendix 1. This nonparametric approach concurs with the GLM, reinforcing the relevance of these factors and providing a more nuanced understanding of their impact on phenotype differentiation.

**Figure 4.**
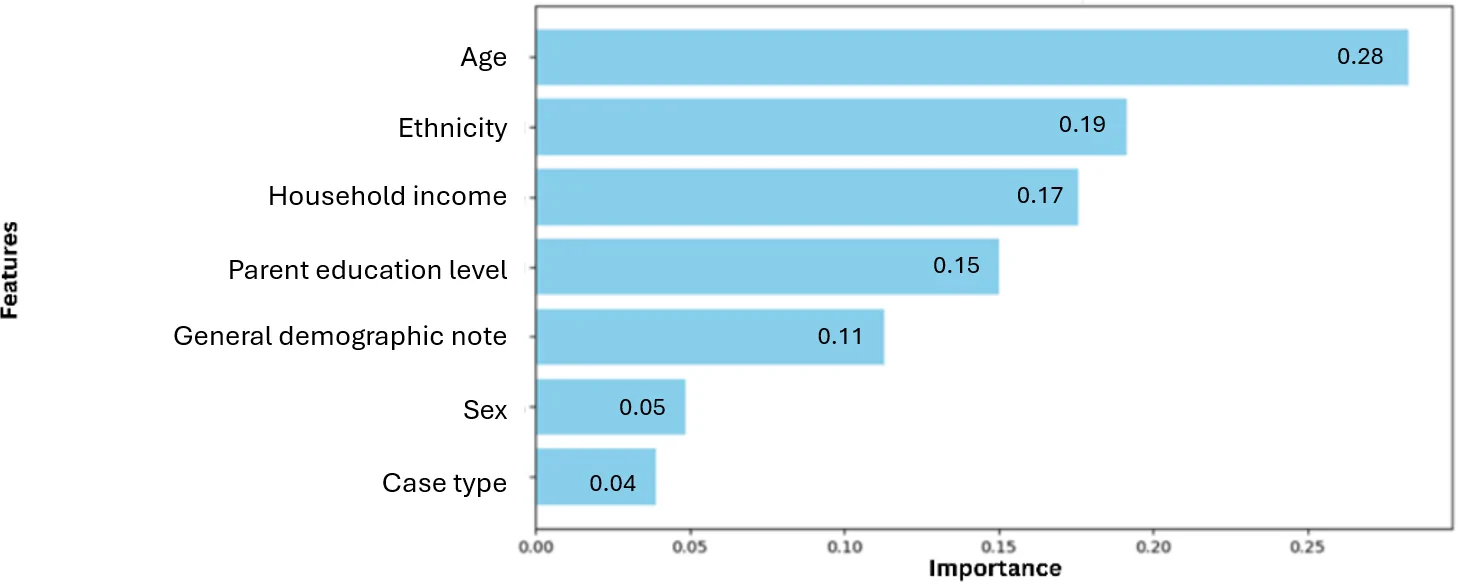
Random forest feature importance scores (mean decrease in impurity from scikit-learn) for Social determinants of health (SDoH) as predictors of phenotype membership. Importance values are nonnegative and normalized to sum to 1 across all features and reflect each variable’s proportional contribution to separating patients into distinct phenotype classes at baseline.

## Discussion

### Principal Results

Compared with the standard PhenoBench framework, we identified and verified 4 distinct pediatric TBI phenotypes characterized by unique injury severity profiles and recovery trajectories, as detailed in the *Results* section (Figure S1 in Multimedia Appendix 1). A GLM analysis confirmed significant SDoH associations for 2 of the 4 phenotypes, a finding corroborated by the random forest model (Figure 4). Of these, clusters 0 and 2 carry the most notable clinical implications. Cluster 0 exhibited a significant positive association between age group and race/ethnicity (*Results*), suggesting that these demographic factors jointly shape phenotype membership even in presentations with a physiologically stable GCS. Cluster 2 showed the strongest SDoH associations overall, with race/ethnicity and its negative interaction with age group both reaching statistical significance, underscoring compounded demographic vulnerability in the most neurologically compromised group. The differential recovery trajectories across all 4 clusters, detailed in the *Longitudinal Analysis of Clusters Over Recovery*, collectively indicate that clinical management must extend beyond acute injury care to address long-term psychological, neurological, and developmental needs, particularly in demographically at-risk pediatric populations.

### Limitations

Most notably, the representativeness of this study is constrained by the exclusion of patient records with missing or illegible data values in CHAI. Additionally, not every patient’s information was available across all data files, which further reduced the size of the dataset used for analysis. Working with this limited dataset may have introduced bias and limited the generalizability of our findings. While the remaining dataset was carefully preprocessed for consistency, it reflects the challenges inherent in working with incomplete health care records. Because patients were retained based on data completeness across 5 feature domains (AIS, vital signs, comorbidities, complications, and GCS) rather than CHAI’s original recruitment criteria [15], the resulting patient subset may not preserve the demographic and injury severity distribution of the full CHAI cohort. While excluding records with missing or illegible values may have introduced bias, this was treated as an assumption rather than formally tested. For methodological rigor, a direct comparison of included vs excluded patients on age, sex, race/ethnicity, and injury severity would substantiate the claim that our analytic sample remains representative of the broader CHAI and pediatric TBI population, consistent with the postexclusion representativeness checks recommended in the cohort-design literature [14].

In addition, the GCS, though widely used to assess the severity of TBI, has certain limitations, particularly in pediatric populations. It may not accurately capture subtle neurocognitive impairments or the evolving nature of TBI recovery, as it focuses primarily on consciousness and motor responses. Moreover, GCS scores provide only a snapshot of injury severity at a given time and may not reflect long-term cognitive, behavioral, or emotional challenges that can arise later in recovery. This limits the scale’s utility in fully understanding complex recovery trajectories in children, necessitating complementary assessments to capture mental health and quality-of-life outcomes.

At last, our analysis adopted several assumptions due to inherent data gaps. We assumed that the conditions under which the CHAI dataset was collected were representative of real-world scenarios to the best of our knowledge. However, such an assumption may introduce biases, given the variability in data collection protocols, missing contextual information, or unrecorded variables. Moreover, the scales used, such as GCS and injury severity measures, may not accurately capture the true level of pain and other symptoms experienced by the patients, especially since children may not always accurately convey their symptoms. Additionally, factors such as the order of underlying injuries, prior medications, and genetic predispositions were not considered in this study. Exploring these variables presents an important area for future research to enhance the understanding of pediatric TBI recovery.

### Knowns, Unknowns, and Misconceptions of Pediatric TBI and SDoH

Pediatric TBI is relatively understudied compared with adult TBI studies. There is a significant gap in understanding the specific impact of TBI on children and adolescents. According to the Centers for Disease Control and Prevention (CDC), children in the United States live with long-term disabilities due to TBI, with the highest incidence among children aged 0 to 4 and 15 to 19 years [20]. Infants are particularly susceptible to abusive TBI, often referred to as “shaken baby” syndrome, which can go unnoticed and undiagnosed due to the subtlety of early symptoms. Children in older age groups, meanwhile, are more susceptible to TBIs from playing contact sports or engaging in risky behaviors, including violence. Statistics indicate that nearly 21% of TBIs among children and adolescents result from sports-related injuries, with contact sports like football and soccer accounting for a significant portion.

Recovery trajectories for children vary drastically from those of adults because brain damage sustained during formative years has lasting impacts on both physical and mental development. Children’s recovery depends heavily on socioeconomic factors such as access to external emotional support systems, therapy, medications, insurance coverage, and the affordability of health care. Unlike adults, who tend to have fully developed resilience and coping mechanisms, children are vulnerable to the negative effects of inadequate care or support systems [21].

A common misconception about pediatric TBI is that children are more resilient due to their neuroplasticity, implying that they can “bounce back” from brain injuries more easily than adults. However, this overlooks the long-term developmental challenges that many children face after sustaining TBIs. While the immediate effects of the injury may not always be severe, cognitive, behavioral, and emotional issues often emerge later, underscoring the importance of continuous monitoring and intervention throughout childhood and adolescence [22].

Prior work of SDoH in pediatric TBI recovery underscores the substantial influence of external support systems, insurance types, income, and other socioeconomic factors in shaping recovery outcomes. For example, access to public insurance and robust social support networks, such as community involvement and family advocacy, have the potential to mitigate the challenges associated with lower socioeconomic status. The Affordable Care Act Medicaid expansion, increased insurance coverage, and enhanced access to rehabilitation services among young adult trauma patients have contributed to reducing socioeconomic disparities in rehabilitation access and mortality disparities between Black and White patients [23]. These interventions complement medical treatments by providing essential mental support, which is crucial for sustained recovery. However, it is noteworthy that this positive outcome was observed only in a minority of cases.

The differential effect of public vs private insurance on patient recovery was not confirmed from this study (see the consistent distribution of insurance types across the phenotypes; Figure S4 in Multimedia Appendix 1). This contradicts the literature indicating that families with private insurance often have greater access to continuous and specialized care, which can be more challenging for lower-income families relying solely on public insurance [24]. Understanding the systemic benefit of stable employment and private insurance on access to specialized care and extended rehabilitation services warrants further investigation.

Children’s geographical location is also known to influence injury severity and long-term patient outcomes [25]. This finding is potentially confounded by insurance type, as public insurance can cover initial medical costs but often lacks the comprehensive support necessary for long-term rehabilitation in TBI cases. Additionally, this inequity predominantly affects female individuals and children of certain ethnic groups [6], as without sufficient community or family support, these patients are more likely to experience prolonged or incomplete recoveries, such as poor mental health.

One of the striking findings revealed in Figure 3 was a consistent decline in mental health outcomes among pediatric patients with TBI. This finding is in line with the clinical recommendation that addressing mental health challenges early is crucial to preventing long-term deterioration, particularly in lower-income families that may face greater challenges in accessing psychological and emotional care services [26].

Overall, despite the complex interplay between clinical outcomes and SDoH, incorporating SDoH screening into pediatric TBI care is essential. A collaborative approach involving health care providers, social workers, educators, and community organizations can ensure that children, especially those from lower-income households, receive the support they need. Public health policy must focus on reducing disparities by expanding rehabilitation services, integrating SDoH assessments, and ensuring that insurance covers both physical and mental health services. Leveraging technology can further enhance care by identifying at-risk children and guiding targeted interventions. Ultimately, a holistic, equitable approach is necessary to improve outcomes, requiring coordinated efforts across health care systems, policymakers, and communities.

### Value of Our Findings on Pediatric SDoH

Although pediatric TBI research has grown substantially over the past decade, the effect of SDoH on a child’s recovery remains underexplored. While prior studies have acknowledged disparities in health care access and outcomes, our study is among the first to directly incorporate socioeconomic factors such as parental income, type of employment, and insurance type into the analysis of recovery trajectories. To the best of our knowledge, this is a novel approach to tracking how these socioeconomic factors influence the course of a child’s recovery, marking an important first step in bridging this research gap.

Furthermore, our study prioritized mental health as a key component of recovery. Unlike many previous studies focusing primarily on physical outcomes, we explicitly included mental health metrics to ensure a holistic understanding of recovery. This reinforces the notion that quality of life, including emotional well-being, is a fundamental aspect of the recovery process. Addressing mental health within the framework of pediatric TBI emphasizes the need for continuous psychological support, recognizing that recovery is not limited to physical rehabilitation but must also encompass behavioral and emotional care.

### Conclusions

This study identified 4 distinct pediatric TBI phenotypes with differing injury profiles, SDoH associations, and longitudinal recovery trajectories. Generalized linear modeling confirmed that 2 of the 4 clusters carried meaningful demographic risk: cluster 0, characterized by adolescents with high injury severity and stable GCS, showed a significant joint effect of age group and race/ethnicity on phenotype membership; cluster 2, the most neurologically compromised group, exhibited the strongest SDoH associations overall, with race/ethnicity and its negative interaction with age group both reaching significance. Clusters 1 and 3 showed no statistically significant SDoH associations, suggesting that factors outside the measured sociodemographic domain (such as injury mechanism or unmeasured comorbidities) primarily drive membership in these groups. These findings were corroborated by a random forest model, reinforcing the robustness of the observed SDoH signal.

Longitudinal analysis revealed divergent recovery trajectories across the 4 clusters, with differential worsening in psychological, neurological, and developmental domains through the 12-month follow-up. Of note, cluster 3, despite presenting with moderate acute injury severity, carried the highest overall impairment burden at all time points, with a sharp escalation in psychological and neurological conditions between 3 and 12 months. This pattern suggests that acute clinical presentation alone is insufficient to predict long-term burden and that psychosocial vulnerability may independently drive trajectory. The 3-month follow-up time point emerged as a critical window for identifying emerging mental health concerns across clusters, indicating that targeted psychological screening at this stage could prevent further deterioration.

Collectively, these findings support the integration of systematic SDoH screening into routine pediatric TBI care. Addressing race/ethnicity-based and age-modulated disparities, particularly in clusters 0 and 2, together with proactive mental health monitoring across all phenotypes are concrete steps that clinical teams can implement. Future work should prospectively validate these phenotypes in external cohorts, examine injury-mechanism–level drivers in clusters 1 and 3, and develop predictive models that combine clinical and SDoH data to flag at-risk children early in the care pathway.

### Translational Impacts of This Study

Children and adolescents face an elevated risk of post-TBI mental health conditions, including anxiety, depression, and post-traumatic stress disorder, with established risk factors including preinjury psychiatric history, female sex, family dysfunction, prior concussions, and ages 10 to 13 years [27]. Despite this, a recent systematic review identified a marked paucity of pediatric-specific evidence for mental health management following moderate-to-severe TBI, with most guidance extrapolated from mild TBI or adult populations [28]. Our cluster trajectories suggest that younger age may be neuroprotective even when acute severity is greater, though this effect appears attenuated among Black/African American and Asian children, as reflected by their lower representation in the neuroprotective cluster. These findings support tailored surveillance stratified by age and race/ethnicity, emphasizing sensorineural and developmental screening in young children and higher-frequency mental health monitoring through adolescence regardless of acute injury severity. Further longitudinal pediatric research is needed to address the persistent evidence gap and confirm the associations reported here.

## Supplementary material

10.2196/87372 Multimedia Appendix 1 Custom clustering using uniform manifold approximation and projection traumatic brain injury and hierarchical density-based spatial clustering of applications with noise clustering (left); clustering via the PhenoBench (right).

## Abbreviations

AIS: Abbreviated Injury Scale
CDC: Centers for Disease Control and Prevention
CHAI: Children’s Health After Injury
DoD: Department of Defense
ED: emergency department
FITBIR: Federal Interagency Traumatic Brain Injury Research
FU12: follow-up after 12 months
GCS: Glasgow Coma Scale
GLM: generalized linear model
HDBSCAN: hierarchical density-based spatial clustering of applications with noise
NIH: National Institutes of Health
PCA: principal component analysis
SDoH: social determinants of health
TBI: traumatic brain injury
