# Supplementary material for: Effect of Social Determinants of Health on Pediatric Traumatic Brain Injury Phenotypes: Retrospective Cohort Analysis
Source: JMIR Pediatr Parent. 2026 Nov 12;9:e87372. doi: 10.2196/87372 (PMC13759174; doi:10.2196/87372)
Supplement: Supplementary file 1 [file pediatrics-v9-e87372-s001.docx]

## Multimedia Appendix

Figure 2. Cluster-wise distribution by Gender: All clusters consistently have twice more male than female patients (for example, Cluster 0 consists of 35% female and 75% male).

Figure 1. Custom clustering using UMAP and HDBSCAN clustering (left); Clustering via the PhenoBench (right).

Figure 4. Cluster-wise distribution by Insurance: All clusters are dominated by Private/Commercial insurance, followed by Medicaid/DSHS, Other specific insurance, Basic Health, and Tricare/CHAMPUS, and this rank order is consistent cluster-wise, with except for Cluster 0 having a slightly more portion of Tricare/CHAMPUS over Basic Health.

Figure 3. Cluster-wise distribution by Household Income: Excluding the most frequent household income bracket (>$100K), which is common across all clusters, Clusters 0 and 2 are characteristic; Cluster 0 is most frequented by the 2^nd^ lowest-income bracket ($20K-40K), whereas Cluster 2 is scarce with the middle-income bracket ($40K – 60K).

Figure 5. Cluster-wise distribution by Race/ethnicity: All clusters are dominated by White ethnicity, followed by Other/Mixed, Hispanic, Black, and Asian, except for Cluster 2 having slightly more Asian over Black.

Figure 6. Cluster-wise distribution by Employment status: All clusters are commonly dominated by Working for Pay, followed by Homemaker categories, but the rest of less common categories show some differences.

Figure 7. Partial impairment scores (Total Score) specific to psychological and behavioral issues, for each cluster across three different timepoints (BL, FU3, FU12): *Cluster 3* dominates (BL = 376, FU3 = 401, FU12 = 563), showing a sharp increase by FU12, reflecting worsening or persistent emotional and behavioral concerns. *Cluster 0* ranks second (164 - 242), while *Clusters 1 and 2* show much lower totals (≈ 60 - 140). The broad error bars (± SD ≈ 100 - 160) highlight considerable between-cluster variability, emphasizing how recovery trajectories diverge most markedly in Cluster 3 over time.

Figure 8. Partial impairment scores (Total Score) specific to developmental and sensory impairments, for each cluster across three different timepoints (BL, FU3, FU12): *Cluster 3* shows the highest overall impairment (BL = 220, FU3 = 179, FU12 = 181), followed by *Cluster 0* (126 - 137), while *Clusters 1 and 2* remain substantially lower (≈ 45 - 60). Scores decrease slightly by FU3 and FU12, suggesting moderate improvement or stabilization. The large error whiskers (± SD ≈ 60 - 80) indicate strong variability in total impairment across clusters, with Cluster 3 contributing most to this spread.

Figure 9. Partial impairment scores (Total Score) specific to neurological and physical conditions, for each cluster across three different timepoints (BL, FU3, FU12): *Cluster 3* shows the highest total neurological and physical condition scores across all timepoints (BL = 99, FU3 = 135, FU12 = 166), indicating a heavier burden of chronic pain, sleep disturbances, and epilepsy relative to other clusters. *Cluster 0* displays moderate values (60 - 76), while *Clusters 1 and 2* remain the lowest overall, suggesting milder neurological complications. The wide whiskers (± SD ≈ 40 - 60) reflect strong variability between clusters, with the gap widening notably by FU12 as Cluster 3’s impairments persist or intensify over time.
